# Yessotoxins in Mollusks of the Galician Coast from 2014 to 2022: Variability, Biotransformation, and Resistance to Alkaline Hydrolysis

**DOI:** 10.3390/toxins15110661

**Published:** 2023-11-16

**Authors:** Juan Blanco, Ángeles Moroño, Fabiola Arévalo, Jorge Correa, Juan Pablo Lamas

**Affiliations:** 1 Centro de Investigacións Mariñas (CIMA), Xunta de Galicia, 36611 Pontevedra, Spain; 2 Instituto Tecnolóxico para o Control do Medio Mariño de Galicia (Intecmar), 36611 Pontevedra, Spain; amoronho@intecmar.gal (Á.M.); farevalo@intecmar.gal (F.A.); jcorrea@intecmar.gal (J.C.); plamas@intecmar.gal (J.P.L.)

**Keywords:** yessotoxins, alkaline hydrolysis, seasonality, interspecific variation, interannual variation, biotransformation, habitat

## Abstract

The presence of yessotoxins (YTXs) was analyzed in 10,757 samples of Galician bivalves from 2014 to 2022. Only YTX and 45-OH YTX were found. YTX was detected in 31% of the samples, while 45-OH YTX was found in 11.6% of them. Among the samples containing YTX, 45-OH YTX was detected in 37.3% of cases. The maximum recorded levels were 1.4 and 0.16 mg of YTX-equivalentsg^−1^, for YTX and 45-OH YTX, respectively, which are well below the regulatory limit of the European Union. The YTX and 45-OH YTX toxicities in the raw extracts and extracts subjected to alkaline hydrolysis were strongly and linearly related. Due to the lack of homo-YTX in Galician samples, the effect of alkaline hydrolysis on homo-YTX and 45OH-Homo-YTX was only checked in 23 additional samples, observing no negative effect but a high correlation between raw and hydrolyzed extracts. Hydrolyzed samples can be used instead of raw ones to carry out YTXs determinations in monitoring systems, which may increase the efficiency of those systems where okadaic acid episodes are very frequent and therefore a higher number of hydrolyzed samples are routinely analyzed. The presence of YTX in the studied bivalves varied with the species, with mussels and cockles having the highest percentages of YTX-detected samples. The presence of 45-OH YTX was clearly related to YTX and was detected only in mussels and cockles. Wild populations of mussels contained proportionally more 45-OH YTX than those that were raft-cultured. Spatially, toxin toxicities varied across the sampling area, with higher levels in raft-cultured mussels except those of Ría de Arousa. Ría de Ares (ARE) was the most affected geographical area, although in other northern locations, lower toxin levels were detected. Seasonally, YTX and 45-OH YTX toxicities showed similar patterns, with higher levels in late summer and autumn but lower toxicities of the 45-OH toxin in August. The relationship between the two toxins also varied seasonally, in general with a minimum proportion of 45-OH YTX in July–August but with different maximum levels for raft-cultured and wild mussel populations. Interannually, the average toxicities of YTX decreased from 2014 to 2017 and newly increased from 2018 to 2021, but decreased slightly in 2022. The relationship between 45-OH YTX and YTX also varied over the years, but neither a clear trend nor a similar trend for wild and raft mussels was observed.

## 1. Introduction

Yessotoxins (YTXs) are ladder-shaped polyether compounds that, in most cases, contain two sulfate groups but can contain three or only one [1,2]. Around a hundred derivatives and analogues have been identified in phytoplankton, seawater, or as products of the metabolic activity of bivalves and other organisms [3,4,5,6,7,8,9,10]. The main toxin, yessotoxin (YTX), was discovered by Murata et al. [11] from extracts of the digestive gland of the scallop *Patinopecten yessoensis* while studying the causative agents of diarrheal shellfish poisoning (DSP). It was found to be co-extracted with okadaic acid (OA) and to kill mice by intraperitoneal (i.p.) injection, which led to the suspicion of its involvement in DSP toxicities [11]. YTX, and at least some of its analogs, seem to be heat-stable [12] and seem to be stable under alkaline conditions [13].

To date, no intoxication of humans by YTXs has been reported, but at least some of them have been shown to kill mice by (i.p.) injection and to be cardiotoxic [14], cytotoxic [15], and immunotoxic [16] to mammals or mammal cells. Even though YTXs seem to be nearly non-toxic to mammals by oral administration [17,18,19], the possible cardiotoxic effects led the European Food Safety Authority (EFSA) to recommend 3.75 mg of YTX equivalents kg^−1^ as the maximum allowable level for human consumption of mollusks [12]. This level was included in the European Union regulation [20]. YTX was initially found to be produced by the dinoflagellate *Protoceratium reticulatum* (=*Gonyaulax grindleyi*) in Japan [21], and some analogs were soon found in the same species [4,5,22]. Currently, it is known that these toxins are produced by several dinoflagellate species belonging to different genera, such as *Lingulodinium polyedrum* [23,24,25], *Gonyaulax spinifera* [26,27], and *Gonyaulax taylori* [28], and that these species, and consequently the YTXs, are worldwide distributed. In Europe, these toxins are frequently found in the Mediterranean Sea [7,9,25,29,30,31,32,33,34,35,36], but their incidence in the Atlantic area seems to be much more limited [37,38,39,40,41].

On several occasions, YTXs, or blooms of YTX-producing species, have been associated with adverse effects on the marine fauna. That was the case of abalone [42] and other marine fauna [43,44,45] in South Africa, Manila clams in Washington State (USA) [46], oyster larvae in British Columbia (Canada) [47], and another abalone in California (USA) [48], and probably also in other organisms (compiled by Landsberg [49]).

**Figure 1:**
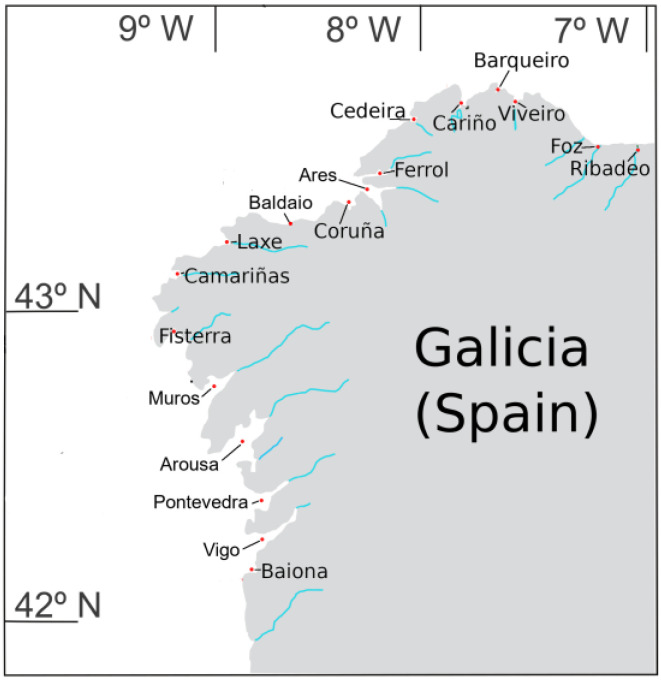
Geographical localization of the areas where the samples were collected (NW Spain).

In Galicia (NW Spain, Figure 1), the EU-regulated lipophilic toxins, which include four YTXs (YTX, 45-OH YTX, homo-YTX, and 45-OH homo-YTX), have been routinely monitored in bivalve mollusks by LC-MS/MS since 2014. Most of the analytical efforts are focused on the toxins of the okadaic acid group, which entails the highest risk in the area [37], and for regulatory purposes, the analysis is performed on extracts subjected to alkaline hydrolysis (to quantify the main toxins and their derivatives). All other regulated toxins (YTXs and azaspiracids (AZAs) among them) and some emergent toxins are analyzed using raw extracts of a subset of the obtained samples. In a previous study on lipophilic toxins, we studied the YTXs in the area from 2014 to 2017, detecting only YTX and 45-OH YTX and establishing a decreasing trend in their toxicities [37]. However, several strains of *P. reticulatum* from the area have been shown to produce small proportions of non-EU-regulated analogs [50], and a strain isolated from the Mediterranean coast of Spain contains homo-YTX as the main toxin [50], suggesting that the toxin profile deserves additional investigation. In this work, we have confirmed the main sources of YTX variability in the area (Figure 1), checked the previously detected temporal trend, and evaluated the possibility of using extracts subjected to alkaline hydrolysis to quantify the toxicities of the regulated yessotoxins, which would make it possible to optimize those monitoring systems without the presence of AZA toxins.

## 2. Results

### 2.1. General

From 3 January 2014 to 28 December 2022, 10,757 samples were analyzed for the presence of yessotoxins. Only YTX and 45-OH YTX were detected in these samples. YTX levels above the detection limit of the method were recorded in 3339 samples (31%) and 45-OH YTX in 1246 samples (11.6%). The 45-OH YTX was detected in 37.3% of the samples containing YTX. The maximum levels were recorded on 18 August 2014 in Ría de Ares (ARE): 1.43 mg YTX-eq kg^−1^ and 0.16 for YTX and 45-OH YTX.

### 2.2. Hydrolisable Derivatives and Quantification after Alkaline Hydrolisis

**Figure 2:**
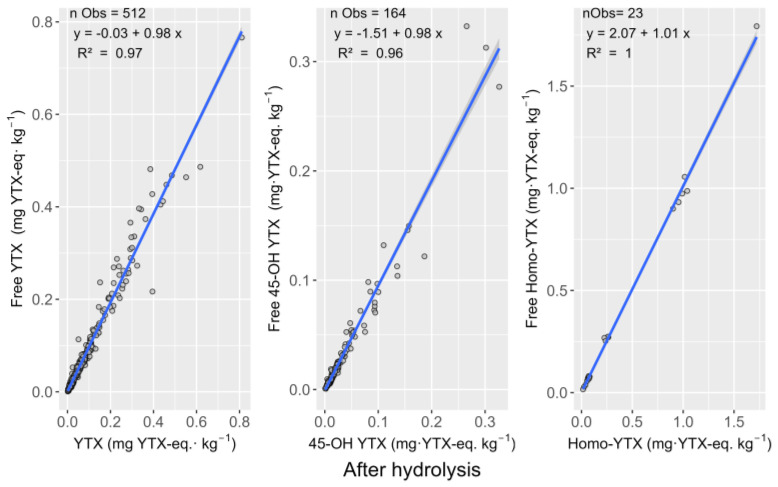
Relationship between the toxicities of YTX, 45-OH-YTX, and Homo-YTX in non-hydrolyzed (free toxin) and hydrolyzed (after hydrolysis) extracts. The top lines show the number of samples included in the regression (nObs), regression equation, and corresponding determination coefficient (R^2^).

The toxicities of YTX and 45-OH YTX before and after hydrolysis were strongly and linearly related (Figure 2). The regressions of the toxicities of each toxin in its free form (non-hydrolyzed extracts) on those of the total toxin (free OA + hydrolysable derivatives measured in the hydrolyzed extracts) were very good, with R^2^ and slopes close to 1 (Figure 2).

No samples containing Homo-YTX were available from the area, but the effect of hydrolysis was checked in 11 bivalve samples from intercomparative studies and 12 samples prepared by spiking bivalves with a Homo-YTX reference solution. In that case, the R^2^ obtained for the regression of the results of the quantification in the raw on those of the hydrolyzed extracts was 0.998, and the slope was 0.989 (Figure 2).

### 2.3. Interspecific Variation

**Figure 3:**
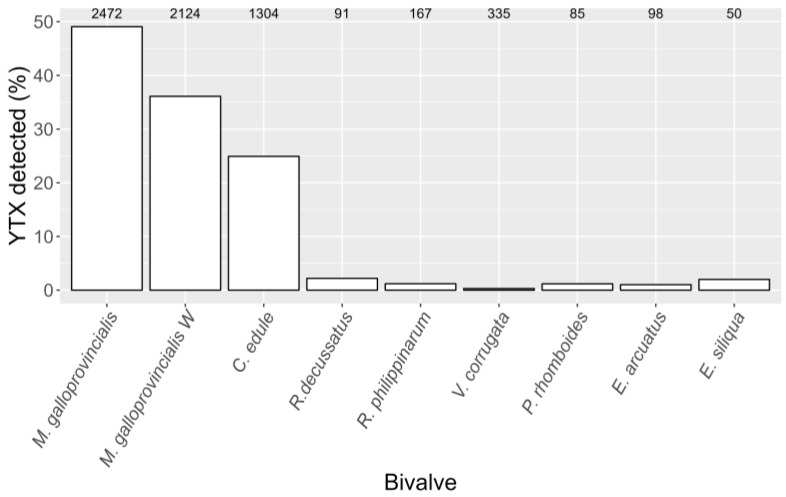
Percentage of the analyzed samples in which YTX was detected in the studied species and mussel habitats (*M. galloprovincialis* = raft-cultured mussels and *M. galloprovincialis W* = wild mussels). The numbers at the top are the total number of samples analyzed.

The accumulation of YTX in the studied bivalves was species-dependent. The highest percentages of YTX-samples in which YTX was detected were observed in mussels (*M. galloprovincialis*), both raft-cultured and wild populations, and cockles (*C. edule*), with percentages of presence in samples between 49.1% and 24.9% (Figure 3). In all clams (including razor clams), the percentage of samples in which YTX was detected was much lower, with a maximum value of 2.2% recorded in *R. decussatus* (Figure 3).

**Figure 4:**
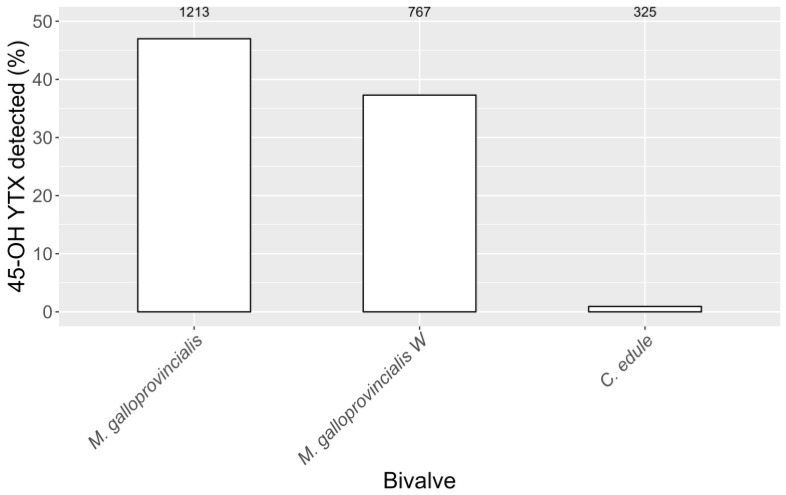
Percentage of samples with YTX that also have detectable levels of 45-OH YTX. The numbers at the top represent the total number of samples in which YTX was detected (*M. galloprovincialis* = raft-cultured mussels and *M. galloprovincialis W* = wild mussels).

Furthermore, 45-OH YTX was only found in the three bivalve species with the highest incidence of YTX (except two species in which it was detected in less than three samples). Moreover, 45-OH YTX appeared only when YTX was present, being detected in 47%, 37.3%, and 0.9% of samples with YTX in raft mussels, wild mussels, and cockles, respectively (Figure 4). Clearly, the percentage of 45-OH YTX detections in cockles (only three positive samples) was substantially lower than that in mussels.

**Figure 5:**
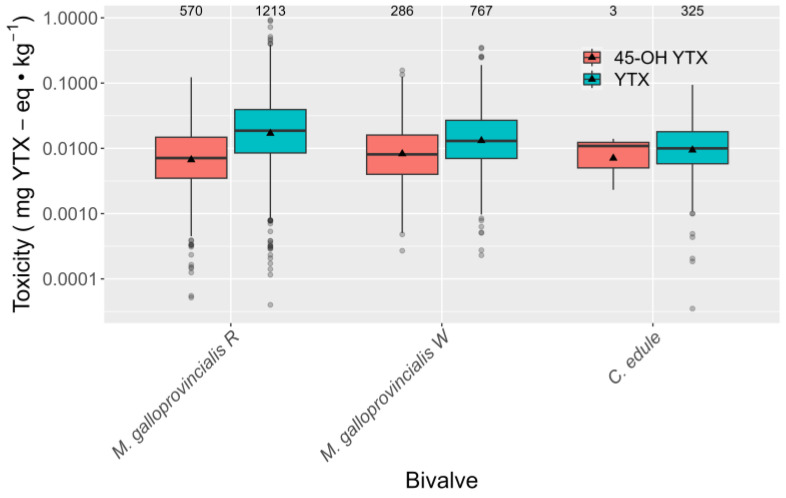
YTX and 45-OH YTX toxicities in the studied species, in which YTX was detected in more than two samples. The upper and lower lines of the boxes represent the first and third quartiles, and the middle line is the median. Vertical lines extend to 1.5 times the interquartile range for the corresponding quartile. Circles represent the observations that fall outside the range defined by the extremes of the vertical lines (outliers). The numbers at the top represent the number of observations.

In general, the average toxicities of YTX were higher than those of 45-OH YTX in all the bivalve species studied, with the largest difference being detected in raft mussel, followed by wild mussel and cockle, which had the minimal difference (with only three detections of 45-OH YTX) (Figure 5).

### 2.4. Relationship between YTX and 45-OH YTX

**Figure 6:**
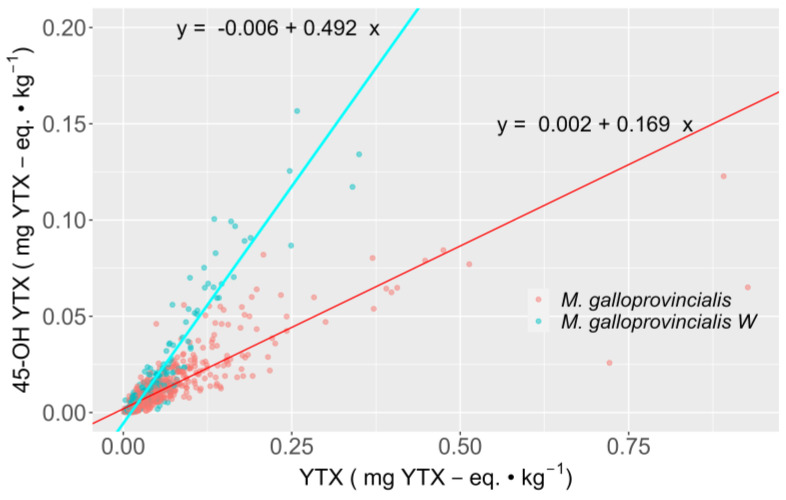
Regression lines of the toxicities of 45-OH YTX as a function of the toxicities of YTX and the mussel habitat (raft = *M. galloprovincialis* and wild = *M. galloprovincialis W*).

The relationship between the two toxins could only be studied in mussels because of the low number of simultaneous detections in all other species. The relationship was approximately linear, but with statistically significant different regression slopes for wild and raft mussels. In general, wild mussels have more 45-OH YTX in relation to YTX than raft-cultured mussels do. In fact, the slope of the regression line corresponding to wild mussels was 2.9 times larger than that corresponding to raft mussels (Figure 6).

### 2.5. Spatial Variation

#### 2.5.1. Toxicities

**Figure 7:**
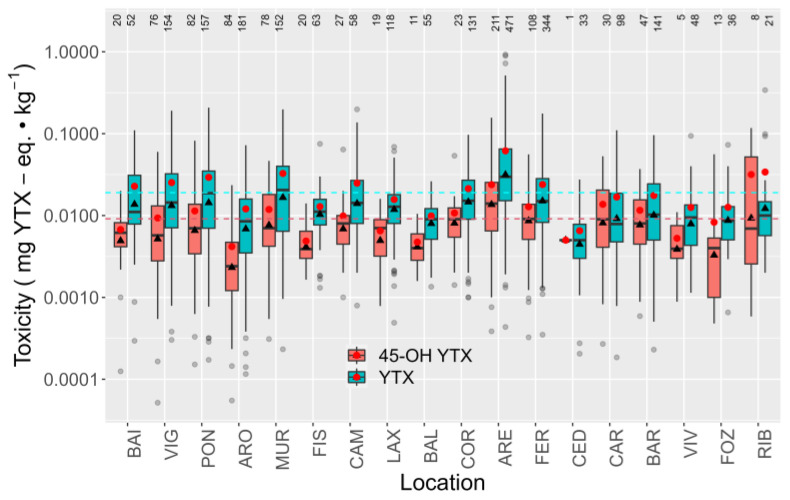
Toxicities of YTX and 45-OH YTX in Galician Rías. Boxes show the interquartile ranges (IQR) of the observations and the median (central horizontal line). The vertical lines extend from the first and third quartiles to the maximum or minimum observations, which do not exceed 1.5 times the IQR. The more extreme observations are considered outliers and are represented as empty circles. Triangles and red dots represent geometric and arithmetic means, respectively. The numbers at the top are the total number of samples analyzed.

Both YTX and 45-OH YTX toxicities varied with the sampling location. The ría of Ares (ARE) was the area most affected by these toxins (Figure 7). The locations to the north of Ferrol (FER), which include those in the Cantabrian Sea, attained substantially lower toxin levels than the southernmost locations. In general, the spatial trends were similar for the two toxins. In the few locations in which the two trends did not coincide, the number of 45-OH YTX detections was low.

#### 2.5.2. Relationship between YTX and 45-OH YTX

**Figure 8:**
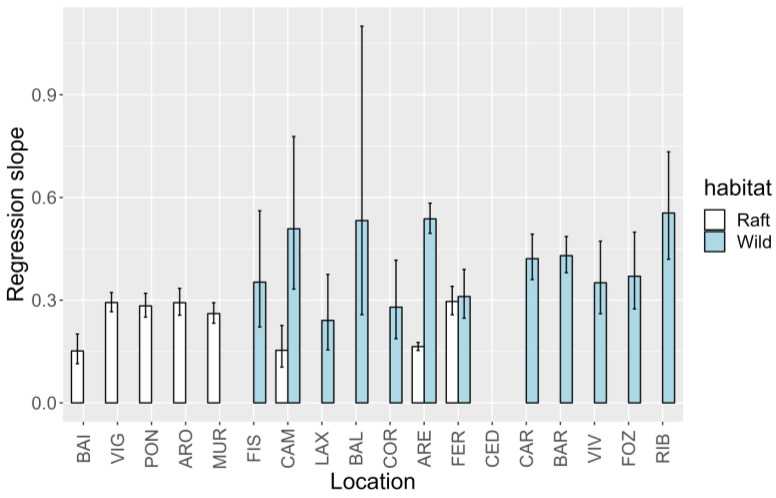
Slopes and their errors in the regressions between 45-OH YTX and YTX at different sampling locations and mussel habitats.

The relationship between the two YTXs varied with the location where the samples were collected, both in wild and raft-cultured mussels. For raft-cultured mussels, the slopes of the regression between 45-OH YTX and YTX ranged from 0.15 to 0.30. The lowest values were recorded in Baiona (BAI), Camariñas (CAM), and Ares (ARE) (Figure 8). For all other locations, the slopes were very similar and close to the recorded maximum. For wild mussels, the slopes ranged from 0.24 to 0.55. The locations from the north coast (from Cariño to Ribadeo, CAR to RIB), in general, were more homogeneous and, on average, had higher slopes than the locations on the west coast, which presented an apparently random distribution of locations with high and low slopes. Surprisingly, in the few locations in which the two types of mussels were sampled (CAM, ARE, and FER), the magnitudes of the slopes were inverted when compared to mussels of the same type in other locations. Thus, these three locations are among the ones with the lowest slopes for raft mussels but among the locations with the highest slopes for wild mussels.

### 2.6. Seasonal Variation

#### 2.6.1. Toxicities

**Figure 9:**
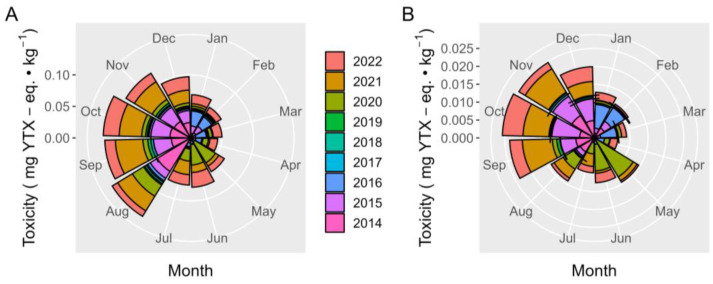
Seasonal variation of YTX (**A**) and 45-OH YTX (**B**) toxicities.

The YTX and 45-OH YTX toxicities showed a clear seasonal trend, with high average levels from September to December for the two toxins (Figure 9). YTX levels were maximal in August, but they did not correspond to 45-OH YTX, in which a relatively low average toxicity was detected. The December toxicities were also lower than those of the preceding months but higher than those of the first half of the year.

#### 2.6.2. Relationship between YTX and 45-OH YTX

**Figure 10:**
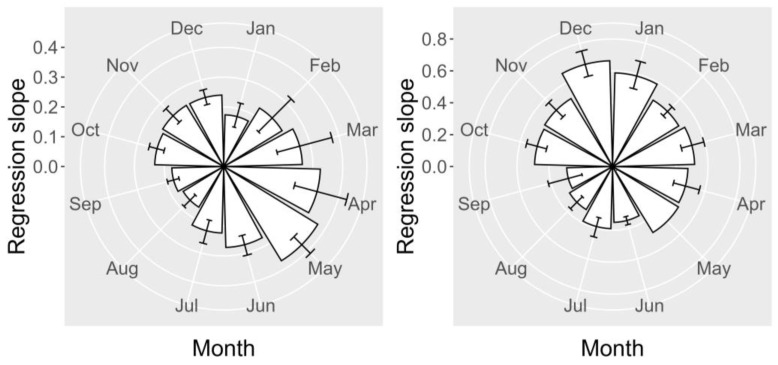
Seasonal variation of the slopes of regression between 45-OH YTX and YTX in raft (**left** panel) and wild mussels (**right** panel).

The proportion of 45-OH YTX in relation to YTX in raft mussels varied throughout the year, with the regression slope increasing towards spring, reaching a maximum in May, and decreasing afterwards until August, when it started to increase until intermediate levels, which were maintained in autumn and winter (Figure 10).

In wild mussels, the relationship between these two toxins was different. The minimal slopes, as in raft mussels, were recorded in summer (September and August); however, the maximum slopes were observed in winter (December and January) instead of spring (Figure 10).

### 2.7. Interannual Variation

#### 2.7.1. Toxicities

**Figure 11:**
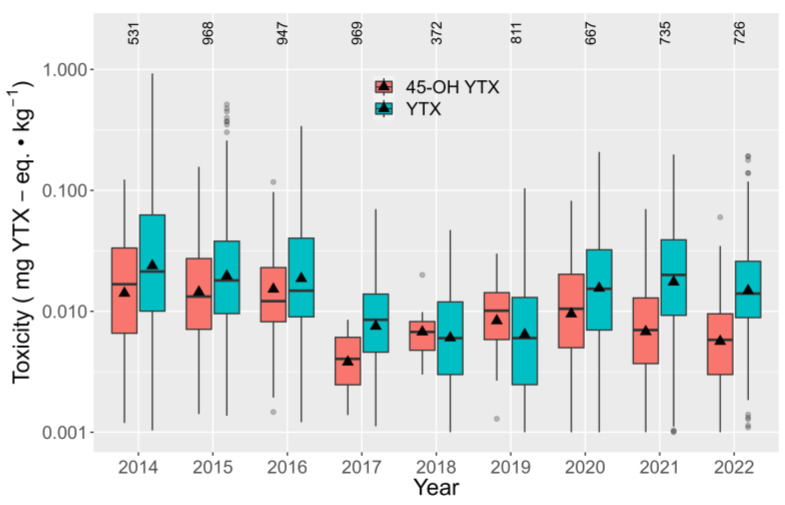
Interannual variation of YTX and 45-OH YTX toxicities in the studied area. All symbols are as in Figure 5.

During the period of study, the average toxicities of YTX decreased from 2014 until 2017–2018, but since that period, they increased for the next three years and slightly decreased in 2022 (Figure 11). Regarding the interannual trend for 45-OH-YTX, it showed a similar trend to YTX but with an earlier increase and final decrease.

#### 2.7.2. Relationship 45-OH YTX YTX

**Figure 12:**
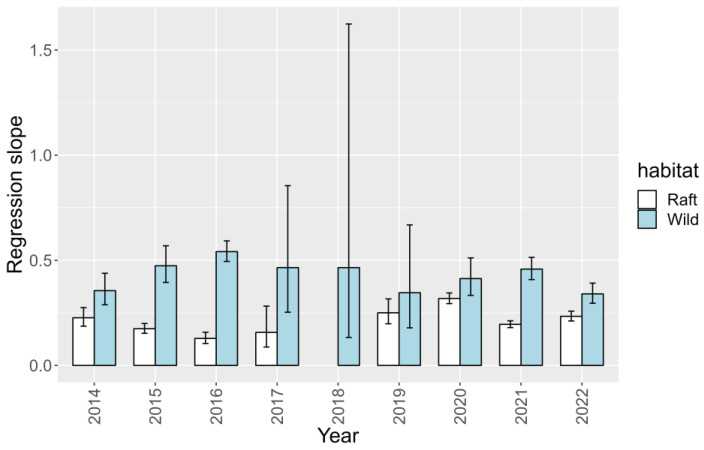
Slopes and their errors in the regressions between 45-OH YTX and YTX during the studied years in the two mussel habitats (wild populations and raft cultures).

The relationship between 45-OH YTX and YTX in raft mussels was not constant throughout the period of study. The slope of the regression decreased until 2018, concurrently with the decrease in YTX average toxicities (Figure 12). After 2018, no trend could be detected.

In wild mussels, the relationship between the two toxins during the studied period was very different from that observed in raft-cultured mussels (Figure 12). Two maxima were found in 2016 and 2021, and an intermediate minimum at 2010, contrasting with the maxima at 2014 and 2020 and the minimum at 2018 found in raft mussels.

## 3. Discussion

### 3.1. Toxin Profile and Possible Use of Hydrolyzed Extracts to Quantity YTX

For the YTX group, only YTX and 45-OH YTX were detected in the analyzed samples. This is consistent with our previous work [37] and, together with the mollusk profiles found in Scotland [51] and in several dinoflagellate strains from Galicia [24,50,52], Andalusia (Spain) [24], Portugal [53], Scotland [54], Helgoland (Germany) [55], and Norway [56], suggests that it could be a common profile in a large part of the Atlantic coast of Europe. In Norway, notwithstanding, the estimations of YTX toxicities were higher when obtained by ELISA than when obtained by LC-MS, indicating that some non-identified analogs could be involved [57], and in Sweden, some *L. polyedrum* strains do not contain YTX but other analogs [3]. The estimated toxicities of both YTX and 45-OH YTX obtained after alkaline hydrolysis of the methanolic extracts were very highly correlated with those obtained in raw methanolic extracts. Moreover, the slope of the regression of raw vs. hydrolyzed estimates was very close to 1, indicating that, if there were hydrolysable derivatives of the regulated toxins, they would be present in a very small percentage. This low contribution of derivatives to the total amount of YTXs could be expected from the toxin profiles of some *P. reticulatum* strains from the Atlantic coast of Spain [50,58]. Even when no homoyessotoxin was found in any Galician sample, the results obtained with samples from proficiency tests indicate that hydrolyzed extracts can be used to quantify all the regulated YTXs reliably. In geographical areas where AZA toxins are not present, the possibility of using the hydrolyzed extract to quantify OA’s group and YTX´s group would make the monitoring system more efficient. In a Galician-specific case where the presence of OA toxins above the legal limit is frequent and recurrent, a significantly higher number of samples are analyzed after alkaline hydrolyses than without hydrolysis; therefore, it is a significant improvement to obtain accurate information about YTXs in the hydrolyzed samples. Nowadays, according to European regulation [59], during harvesting periods, at least with a weekly frequency, it is necessary to inject two extracts per sample: (1) hydrolyzed extract (total content of OA/DTX toxins) and (2) raw extract (YTXs and AZAs). According to Article 61, Point 3 of the Commission Implementing Regulation (EU) 2019/627, “Results suggesting an accumulation of toxins in live bivalve mollusk flesh shall be followed by intensive sampling” [59]. In this sense, in geographical areas where the presence of OA toxins above the legal limit is frequent and recurrent, in the absence of AZA episodes, a significantly higher number of samples are analyzed after alkaline hydrolyses than without hydrolysis; therefore, obtaining accurate information about YTXs in the hydrolyzed samples would be a significant improvement”.

### 3.2. YTX Variability among Bivalve Species

As was the case in our previous study [37], none of the analyzed samples had YTX toxicities above the regulatory limit of 3.75 mg YTX-eq kg^−1^, but the percentage of samples in which these toxins were detected was higher than that previously reported. The maximum attained levels were still far from the regulatory threshold (between one third and a half of that level). Mussels were the most affected species by YTXs, followed by cockles. Both presence and toxicity levels in all other species were very low, confirming our previous observations. The detection of 45-OH YTX was practically restricted to mussels, supporting the suggestion of Yasumoto and Takizawa [60] that mussels have a much higher YTX biotransformation capability than other bivalve species. Raft mussels had higher YTX toxicities than wild mussels, but the opposite happened with the levels of 45-OH YTX, thus confirming the pattern found in our previous study. It is also clear that wild mussels contain more 45-OH YTX in proportion to YTX than raft mussels at all YTX toxicities, which suggests that they have different capabilities of YTX biotransformation. The causes of this different capability are unknown. Raft and wild mussel populations are genetically very similar [61], as raft cultures are mostly initiated using juvenile mussels taken from wild populations. Consequently, the observed biotransformation differences should originate from environmental factors. The main difference between the two populations is that the wild mussels live in the intertidal zone, therefore being subjected to periodic emersion, while this does not happen to raft mussels. This process, however, is unlikely to have a direct effect because of the differences in the seasonal patterns of the proportion between the two toxins in the two populations. However, the environmental conditions to which mussels are subjected during emersion could contribute to explaining the observed differences. Montagnac et al. [62] did not find any difference in the scope for growth between wild and cultured bivalves in Prince Edward Island (Canada) using bivalves of the same age, and no important difference in the reproductive cycle was found by other authors [63]. Notwithstanding, some differences in the resource allocation between wild and cultured mussels of different ages have been reported [64]. In Galician mussels, the age difference between wild and cultured mussels could be important because there is no commercial exploitation of wild populations and the culture cycle is around 18 months [65].

### 3.3. Spatial Variation of YTXs

The highest average (geometric mean) YTX toxicities in the area were recorded from the ría of Ares, followed by the ría of Muros, a pattern that had already been found in our previous study dealing with the period between 2014 and 2017 [37]. The lowest levels were recorded from the rías of Cedeira and Arousa. The fact that the two rías with the highest toxicities are adjacent to the two with the lowest ones (or nearly adjacent) suggests that the local factors have a special relevance, as it could be expected for toxins produced by species that produce long-lasting resistance cysts like *Lingulodinium polyedrum* or *Protoceratium reticulatum*. The maximum toxicities of *L. polyedrum* cysts in Galicia have been reported from the area of maximum incidence of YTXs (ría of Ares), but they have also been found in several areas of the Galician coast [66]. *Protoceratium reticulatum* cysts, notwithstanding, were only rarely found in Galician sediments [67], suggesting that the former species might be the main one responsible for the production of YTXs even when *P. reticulatum* is present in Galician waters and has been shown to produce YTXs [52].

The spatial pattern of 45-OH YTX was similar to that of YTX, but the difference between the levels of the two toxins was higher in the locations where all or most samples were raft mussels, as could be expected (Figure 8 and Figure 9).

### 3.4. Seasonality

The highest YTX toxicities in the area took place in late summer and autumn. We do not have available records of the phytoplankton species that can produce these toxins, but the observed pattern coincides with that recorded for the populations of *L. polyedra, P. reticulatum,* and *G. spinifera* in these and geographically close areas [38,39,41,68,69]. During winter, spring, and early summer, the levels are lower and the variability among years is higher. The seasonal pattern is similar for the two toxins, with the main exception of August, when the average level of 45-OH YTX is lower than the one that could be expected from the YTX toxicities. One possible cause for this is that, because 45-OH YTX is a product of YTX transformation in mussels, there is some delay in attaining its maximum levels.

The relationship between the two toxins also varied seasonally. The minimal proportions of 45-OH YTX in relation to YTX (regression slope) were recorded in summer in both raft and wild mussels, which is surprising because the temperature during this period would be expected to favor biotransformation. The difference in the seasonal patterns of the proportion between the two toxins in wild and raft mussels, especially the extended period of high proportion during the winter months for wild mussels, cannot be explained with our current knowledge.

### 3.5. Interannual Variation

The decreasing trend of the average toxicities of YTX with time found in our previous study [37] did not hold in the five subsequent years. In fact, the trend has nearly reverted, increasing from 2018 to 2021 but slightly decreasing in 2022. The 45-OH YTX pattern was similar but not equal. The most striking difference is its low level in 2021, when a local maximum of YTX took place.

The proportions between the two YTXs changed with the years. We do not have any satisfactory explanation for that.

## 4. Conclusions

YTX toxicities in the analyzed samples did not exceed the regulatory limit. YTX and 45-OH YTX were the only toxins detected in the analyzed samples. Appreciable amounts of hydrolysable conjugated forms do not seem to be present because hydrolyzed extracts can be used to quantify YTXs reliably. Mussels were the most affected species by YTXs, followed by cockles, while other bivalve species had very low levels. The presence of 45-OH YTX was primarily restricted to mussels, suggesting different biotransformation capabilities in mussels compared to other bivalve species. The highest YTX toxicities were recorded in the ría of Ares and the ría of Muros, while the lowest levels were found in the rías of Cedeira and Arousa. Local factors appear to play a significant role in YTX toxicity levels. YTX toxicities were highest in late summer and autumn, with lower levels during winter, spring, and early summer. The relationship between YTX and 45-OH YTX varied seasonally, with differences in biotransformation rates during different seasons not yet fully understood. There was a decreasing trend in the average toxicity of YTX in the years leading up to 2017, but this trend did not continue in the subsequent years. Proportions between the two YTXs changed over the years, and there is no clear explanation for this phenomenon.

## 5. Material and Methods

### 5.1. Sampling

From January 2014 to December 2022, 10,757 bivalve samples were collected from diverse Galician mollusk production areas (Figure 1) by Intecmar in the framework of the Galician monitoring system. Sampling frequency was, at least, weekly for each production area. Most samples were obtained from mussel (*Mytilus galloprovincialis*) culture facilities, whereas others were obtained from natural beds of mussels and other bivalve species, such as cockle (*Cerastoderma edule*), clams (*Ruditapes decussatus*, *R. philippinarum*, *Venerupis corrugate,* and *Polititapes rhomboides*), and razor clams (*Ensis siliqua* and *E. arcuatus*). Since mussels are used as sentinel organisms, the natural beds of other bivalve species were sampled only when any of the European Union (EU)-regulated toxins were detected in mussels.

### 5.2. Analysis

#### 5.2.1. Extraction and Hydrolysis

Yessotoxins, together with the other EU-regulated toxins, were extracted from the samples using the standardized operating methods of the EU Reference Laboratory [70]. Briefly, 100–150 g of meat was homogenized, and a 2-g subsample was taken, which was subsequently vortexed twice in 9 mL of 100% MeOH for 1 min and centrifuged at 2000× *g* for 10 min. The supernatants were combined, and the total extract was added to 20 mL with 100% MeOH.

Alkaline hydrolysis of 5 mL aliquots of the obtained extracts was carried out with the following procedure: (1) addition of 625 µL of 2.5 M NaOH; (2) vortexing the mixture for 30 s; (3) heating at 76 ± 4 °C for 40 min; (4) allowing to reach room temperature (usually at least 5 min); (5) checking that there were no solvent losses by evaporation (by weight); (6) neutralizing by adding 625 µL of 2.5 M HCl, vortexing for 1 min (final pH of approximately 7); (7) filtering a 1-mL aliquot through a 0.22 µm syringe filter; and (8) diluting it 5/8 (with 100% MeOH), previously to injection.

All samples were analyzed after alkaline hydrolysis because this step is needed to quantify the toxicities of total okadaic acid and dinophysistoxins (free and esterified forms), which is the main toxin group in the area and, consequently, the main target of the monitoring system. Only a subset of all samples (512 samples) was analyzed without a previous hydrolysis to quantify the remainder of the regulated toxins and some emergent toxins that are less stable at high pH than those of the okadaic acid group.

#### 5.2.2. LC-MS/MS

The LC-MS/MS method used to quantify lipophilic toxins was developed, optimized in-house, and validated following the guidelines in the standard operation procedure of the EU Reference Laboratory [70]. This method was based on those by Gerssen et al. [13] and Regueiro et al. [71] and has UNE-EN ISO/IEC 17025 accreditation (Accreditation N° 160/LE 394).

The chromatographic stationary phase was an Acquity UPLC BEH C18, 1.7 µm, in a 2.1 × 100 mm column (Waters, Barcelona, Spain), which was maintained at 45 °C. Mobile phases A and B were 6.7 mM NH_4_OH (pH11) and MeCN 90% with 6.7 mM NH_4_OH. The elution procedure was as follows: (1) an isochratic step of 25% B (1.66 min); (2) a linear gradient from 25 to 95% B (from minutes 1.66 to 4.3); (3) a new isochratic with 95% B (to minute 6.28); (4) return linearly to 25% B (2 min); and (5) maintenance until the ninth minute to equilibrate the system prior to the next injection.

Three LC-MS systems were used: one Waters Acquity UPCL system (coupled to a Waters Xevo TQ MS) and two Waters Acquity H-class systems (each coupled to a Waters Xevo TQ-S) (Waters, Barcelona, Spain). All MSs were equipped with ESI interfaces and operated in positive and negative ionization modes because other toxins were analyzed simultaneously. YTXs, however, were analyzed in the negative ionization mode. All MS systems shared the following source operating parameters: capillary voltage of 2 kV; solvation temperature of 450 °C; solvation gas flow of 850 L·h^−1^; and cone gas flow of 150 L·h^−1^. The transitions corresponding to the toxins studied are listed in Table 1. In all cases, the precursor ion was [M-2H]^−2^. Yessotoxins were quantified by the external standards method using certificate reference solutions of YTX and homo-YTX obtained from the NRC (Canada) and Laboratorio CIFGA (Spain). The 45-OH analogs of YTX and homo-YTX were quantified by assuming the same response as their non-hydroxylated counterparts and using the same CRMs. Quality controls were carried out by using spiked bivalve samples and freeze-dried Mussel Tissue Certified Reference Material for Multiple Marine Toxins from the NRC (Canada). The limits of detection (LOD) and quantification (LOQ) in bivalve tissues for YTX, 45-OH YTX, and Homo-YTX were 0.001 and 0.06 mg YTX-eq·kg^−1^, respectively. For 45-OH-homo YTX, the LOD is the same, but the LOQ is 0.03 mg YTX-eq·kg^−1^.

### 5.3. Statistical Analysis

All statistical analyses were carried out using R [72]. For general plotting, the R package ggplot2 [73] was used. For model II regression analyses to estimate both the relationship between raw and hydrolyzed samples and between YTX and 45-OH YTX, the smatr package [74,75] was used.

## Figures and Tables

**Table 1:** MS/MS transitions for the analyzed yessotoxins. The first transition for each toxin was used for quantification, and the second was used for confirmation.

Toxin	Precursor Ion (*m*/*z*)	Product Ion (*m*/*z*)	Collision Energy (eV)	Cone Voltage (V)
YTX	570.4	467.4	30	45
		396.3	35	45
45-OH YTX	578.4	467.4	30	45
		396.4	30	45
Homo-YTX	577.5	474.4	30	48
		403.4	30	48
45-OH-homo YTX	585.5	403.4	30	45
		474.4	30	45

## Data Availability

The data are available from Intecmar under request.
